# Multiple Horizontal Transfers of Bacteriophage WO and Host *Wolbachia* in Fig Wasps in a Closed Community

**DOI:** 10.3389/fmicb.2016.00136

**Published:** 2016-02-15

**Authors:** Ningxin Wang, Sisi Jia, Heng Xu, Yong Liu, Dawei Huang

**Affiliations:** ^1^Shandong Provincial Key Laboratory for Biology of Vegetable Diseases and Insect Pests, College of Plant Protection, Shandong Agricultural UniversityTai’an, China; ^2^Key Laboratory of Zoological Systematics and Evolution, Institute of Zoology, Chinese Academy of SciencesBeijing, China

**Keywords:** fig wasps, *Wolbachia*, bacteriophage WO, horizontal transfer, specificity, fig syconia

## Abstract

*Wolbachia*-bacteriophage WO is a good model system for studying interactions between bacteria and viruses. Previous surveys of insect hosts have been conducted via sampling from open or semi-open communities; however, no studies have reported the infection patterns of phage WO of insects living in a closed community. Figs and fig wasps form a peculiar closed community in which the *Ficus* tree provides a compact syconium habitat for a variety of fig wasp. Therefore, in this study, we performed a thorough survey of *Wolbachia* and bacteriophage WO infection patterns in a total of 1406 individuals from 23 fig wasps species living on three different fig tree species. The infection rates of *Wolbachia* and phage WO were 82.6% (19/23) and 39.1% (9/23), respectively. Additionally, phage WO from fig wasps showed strong insect host specificity based on *orf7* sequences from fig wasps and 21 other insect species. Probably due to the physical barrier of fig syconium, most phage WO from fig wasps form a specific clade. Phylogenetic analysis showed the absence of congruence between WO and host *Wolbachia*, WO and insect host, as well as *Wolbachia* and fig wasps, suggesting that both *Wolbachia* and phage WO exchanged frequently and independently within the closed syconium. Thus, the infection pattern of bacteriophage WO from fig wasps appeared quite different from that in other insects living outside, although the effect and the transfer routes of phage WO are unclear, which need to be investigated in the future.

## Introduction

*Wolbachia* (Alphaproteobacteria) are maternally inherited obligatory intracellular symbionts that are found in a wide range of arthropods and filarial nematodes (Stouthamer et al., 1999; Taylor et al., 2005), at rates ranging from 20 to 76% (Hilgenboecker et al., 2008; Fenton et al., 2011). The success of *Wolbachia* in achieving this high prevalence is associated with its ability to induce a variety of phenotypes, from mutualism in nematodes to various reproductive manipulations in arthropods, including cytoplasmic incompatibility (O’Neill and Karr, 1990), parthenogenesis (Stouthamer et al., 1990), male killing (Jiggins et al., 2001), feminization (Bouchon et al., 1998), and even speciation (Rokas, 2000). Bacteriophages are the most abundant organisms in the biosphere (Plett et al., 2011) and play important roles in bacterial genome evolution. The temperate phage WO was first detected in 2000 and is the only one bacteriophage known to infect *Wolbachia* (Masui et al., 2000). Accompanied by the widespread distribution of *Wolbachia*, it was reported that about 89% of *Wolbachia* strains are infected with WO (Bordenstein and Wernegreen, 2004). Polymerase chain reaction (PCR) amplification of the minor capsid gene *orf7* has shown that the phage occurs in the majority of the parasitic A and B *Wolbachia* supergroups (Bordenstein and Wernegreen, 2004; Gavotte et al., 2007). Moreover, most phage-infected *Wolbachia* strains display low numbers of phage types, with 85% showing only one or two different phage types (Gavotte et al., 2007; Tanaka et al., 2009).

The tripartite insect host–*Wolbachia*–phage WO is an ideal model system for studying interactions among viruses, bacteria, and eukaryotes (Bordenstein et al., 2006). The phage WO is the only known mobile genetic element that may transform the genome of *Wolbachia* (Metcalf and Bordenstein, 2012). In *Wolbachia*, prophage regions can comprise more than 20% of mobile DNA genes and account for the largest fraction of absent/divergent genes between closely related strains (Chafee et al., 2010). Some researchers have investigated the relationship between *Wolbachia* and WO in such insect species as *Drosophila simulans*, *Ephestia kuehniella*, *Nasonia vitripennis*, *Culex pipiens*, and *Gryllus pennsylvanicus*; moreover, no phylogenetic congruence between *Wolbachia* and WO has been shown, suggesting that the lateral transfer of WO in *Wolbachia* is not unusual (Masui et al., 2000; Bordenstein and Wernegreen, 2004; Chafee et al., 2010). Thus, unraveling the infection status and evolutionary dynamics of *Wolbachia* and WO may be the key to understanding the interactions among these organisms and methods for exploiting such interactions.

Previous surveys of insect hosts have been conducted via sampling from open and semi-open communities; however, no studies have reported the infection patterns of bacteriophage WO from insects living in a closed community. Thus, it is particularly interesting to consider the infection patterns of WO in fig wasps and the relationship with *Wolbachia* as this system occurs within an enclosed syconia. Figs and fig wasps constitute a well-known system of mutualism (Weiblen, 2002): figs (Angiospermae, Dicotyledoneae, Urticales, Moraceae) are pollinated on their inflorescences by their obligate fig wasps (Insecta, Hymenoptera, Chalcidoidea), and the fig wasps lay their eggs in the figs, wherein the eggs develop (Rønsted et al., 2005). The inflorescences, called syconia, provide fig wasps with a compact habitat that is isolated from the outside world (Schiﬄer, 2002). Besides pollinating wasps, some non-pollinators do not enter the syconia, but inject eggs through the fig wall (Schiﬄer, 2002). Both the seeds and the offspring of fig wasps develop in the fig syconium until the fig reaches maturity. Previous surveys have shown that the incidence of *Wolbachia* in fig wasps is up to 59–67%, markedly higher than that in other insects (Shoemaker et al., 2002; Haine and Cook, 2005; Chen et al., 2010).

The syconia provide fig wasps with a compact habitat, in which *Wolbachia* horizontal transfer has been shown to be more likely to occur in this closed system than in other open and semi-open systems (Yang et al., 2012). Thus, in this study, we sought to determine the bacteriophage WO infection patterns in fig wasps, as well as phage diversity within *Wolbachia* strains and within different fig wasps. We investigated 23 fig wasp species from three fig species to elucidate the phage infection patterns. Furthermore, we want to find whether the horizontal transfer of *Wolbachia* has an effect on the diversity and evolutionary dynamics of the temperate bacteriophage WO.

## Materials and Methods

### Identification of Fig Wasps and DNA Extraction

All fig wasps were collected from fig trees in Hainan Province, China, from 2005 to 2013. We collected fig fruits in period D (late in the fruiting cycle but before the period in which fig wasps emerge) and then cultivated the fruits until the fig wasps emerged. The fig wasps were then collected, deposited in 95% alcohol, and stored at –20°C for later use. Different species were morphologically classified under a Nikon SMZ80 microscope. The fig wasp species identified in this study are listed in **Table 1**.

**Table 1:** *Wolbachia* and bacteriophage WO infection patterns in 23 fig wasps.

Fig	Biology	Fig wasp	Individuals screened	*Wolbachia* infect frequency (%)	*Wolbachia* haplotype number (supergroup)	WO infect frequency (%)	WO type number
*Ficus hispida*	dioecious	•*Ceratosolen solmsi*	130	95	1 (A)	95	1
		*Apocrypta bakeri*	42	0	—	0	—
		*Philotrypesis pilosa*	140	100	4 (A)	100	2
		*Philotrypesis* sp.	76	0	—	0	—
*Ficus auriculata*	dioecious	• *Ceratosolen emarginatusApocryptophagus* sp.*Philotrypesis* sp.1*Sycoscapter* sp.1	13286126140	100100100 100	3 (A)3 (A)4 (A)11 (A)	100100100100	2311
*Ficus benjamina*	monecious	• *Eupristina koningsbergeri*	30	100	3 (A)	100	1
		*Walkerella beniamini*	36	60	2 (A/B)	42	1
		*Walkeralla* sp.n	36	40	3 (A/B)	22	1
		*Sycoscapter* sp.1	36	79	Data were not given	0	—
		*Sycoscapter* sp.2	36	100		0	—
		*Philotrypesis* sp.1	36	58		0	—
		*Philotrypesis* sp.4	36	17		0	—
		*Philotrypesis* sp.5	36	43		0	—
		*Sycophila* sp.1	36	0		0	—
		*Sycophila* sp.2	36	78		0	—
		*Sycophila* sp.3	36	50		0	—
		*Sycophila* sp.4	36	0		0	—
		*Sycobias* sp.1	36	89		0	—
		*Sycobias* sp.2	36	32		0	—
		*Acophila* sp.1	36	35		0	—

*“•” indicates the pollinator of each fig; “—”indicates no signal was detected*.

Total genomic DNA was extracted from each individual sample using EasyPure Genomic DNA Extraction Kits (TransGen, Beijing, China). Initially, genomic DNA was screened for the quality of the template using mitochondrial cytochrome c oxidase 1 (*CO1*) (Chambers et al., 2011; Dyer et al., 2011) and nuclear ribosomal DNA internal transcribed spacer 2 (*ITS2*) (Partensky and Garczarek, 2011) via PCR (Kraemer and Velicer, 2011). Poor quality DNA templates were discarded.

### PCR and Sequencing

The samples were first screened for *Wolbachia* infection by PCR amplification with the primers wsp81F (5′-TGG TCC AAT AAG TGA TGA AGA AAC-3′) and wsp691R (5′-AAA AAT TAA ACG CTA CTC CA-3′), which amplified a portion of the *Wolbachia* surface protein gene (*wsp*) (Zhou et al., 1998). If the amplification of *wsp* did not yield a sufficient band on agarose gels, another two pairs of primers were used, ftsZ-F/R for amplification of the *Wolbachia* cell division gene *ftsZ* and 16SwolF/R for amplification of the *Wolbachia* 16sRNA gene (O’Neill et al., 1992; Jeyaprakash and Hoy, 2000). WO was screened by the primers orf7F (5′-GTC TGG AAA GCT TAC AAA AAG-3′) and orf7R (5′-GCT CTA TAA ATT CTC CTA T-3′), and samples that were negative for *orf7* were rechecked by another two pairs of gene primers, ORF2F/R and WD0633F/R (Masui et al., 2000). ddH_2_O was used as a blank control for all amplifications. PCR amplification was performed in a volume of 25 μL, containing 2.5 μL 10× buffer, 0.2 mM dNTPs, 0.5 μM of each primer, and 0.5 U of Trans Taq Enzyme (TransGen Biotech, Beijing, China).

Polymerase chain reaction products were purified using an EasyPure PCR purification Kit (TransGen) and then sent to Beijing Genomic Institute for sequencing. When multiple peaks appeared, the products were cloned in the pEasy-T5 vector (TransGen), and 3–7 positive clones were picked for sequencing.

### Sequence Analysis

#### Raw Sequence Treatments

Sequence homology analysis was performed with the BLAST program in NCBI Web. Haplotypes are defined as having greater than 1.5% nucleotide diversity in the *orf7* gene (Chafee et al., 2010). *Wolbachia* strains that were not infected by phage WO were not included in our study in order to improve readability. If multiple identical sequences were obtained from each species, we chose only one to represent the species. We reserved different sequences and removed identical sequences, yielding 34 *wsp* sequences and 12 *orf7* sequences. The sequences have been deposited in GenBank under the following accession numbers: KT355405–KT355450.

#### Phylogenetic Analyses

The *wsp* and *orf7* sequences were aligned to relevant sequences previously published on NCBI with Clustal W in BioEdit (Hall, 1999). Maximum likelihood (ML) was carried out to construct the phylogenetic tree using MEGA 6 (Tamura et al., 2013). Model selection for the ML analysis was estimated using the Akaike information criterion in Modeltest v3.7. The DNA substitution model was the general time reversible (GTR) model in which the gamma distribution and invariant sites were estimated from the data (GTR+I+G). ML bootstrap values were generated from 1000 bootstrap replicates.

## Results

### *Wolbachia* Infection Patterns in Fig Wasps

We screened a total of 1406 wasps of 23 species for *Wolbachia* and WO infection. The results are listed in **Table 1**. Nineteen out of 23 fig wasp species were shown to be infected with *Wolbachia*. This infection incidence (83%) was higher than in wider screenings of fig wasps in Panama (59%) and Australia (67%) (Shoemaker et al., 2002). The *Wolbachia* infection incidences in the three syconia in our survey differed, ranging from 100% in *Ficus auriculata* (4/4) to 86.7% in *F. benjamina* (13/15) and 50% in *F. hispida* (2/4). The *Wolbachia*-infected fig wasp species from *F. hispida* and *F. auriculata* showed high infection rates, nearly 100%, which agreed with the reported “most or few” (>90% or<10%) infection pattern within one species (Hilgenboecker et al., 2008). However, except for *Eupristina koningsbergeri* and *Sycoscapter* sp.2, which had 100% infection rates, while *Sycophila* sp.1 and *Sycophila* sp.4 were completely uninfected, most species in *F. benjamina* had moderate infection rates, ranging from 17 to 89%, which is similar to our previous survey in *F. benjamina* (Yang et al., 2012).

**FIGURE 1:**
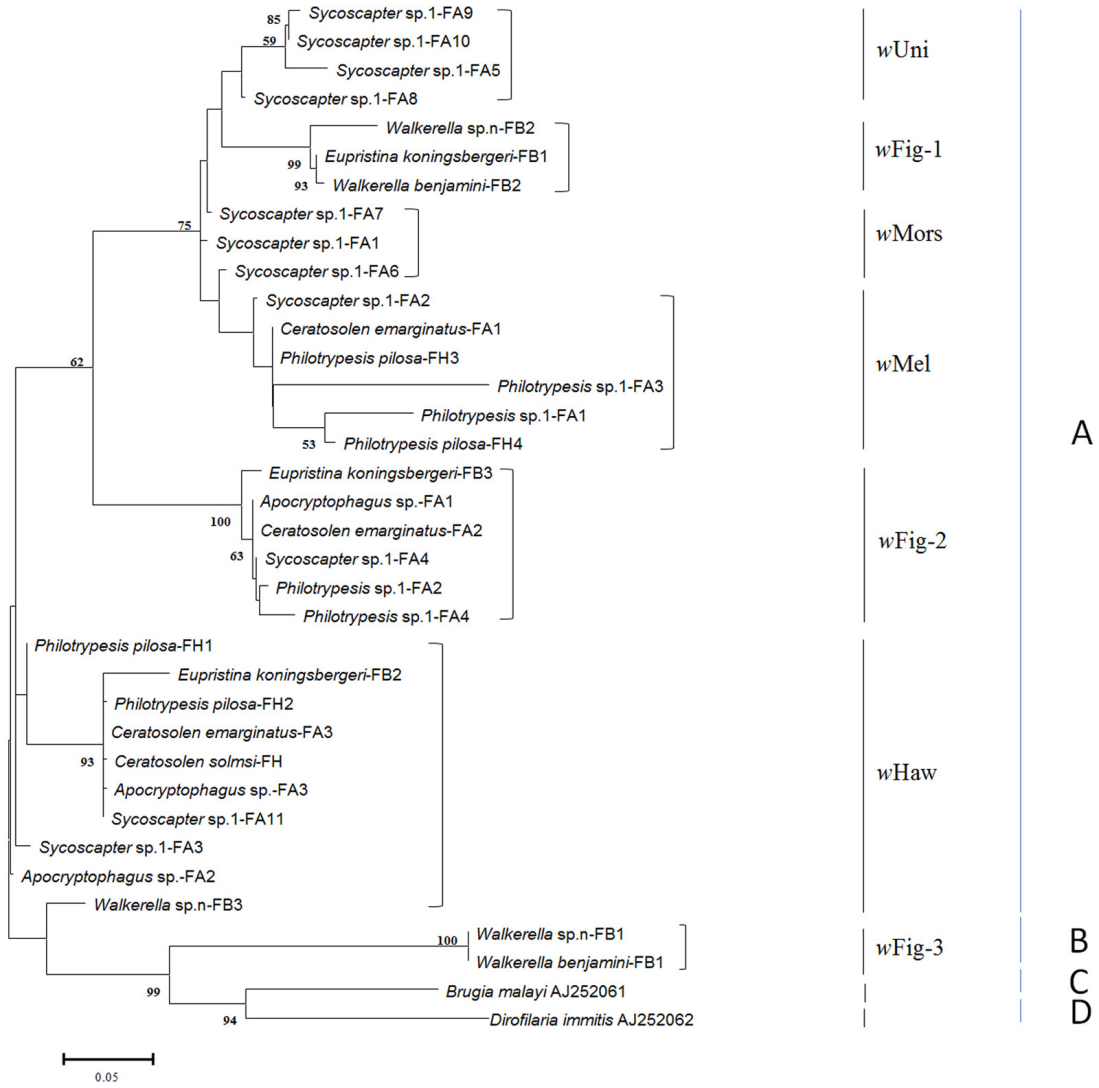
**ML tree constructed with *wsp* sequences from fig wasps**. Two sequences from supergroup C and D are used as outgroups. The supergroups (A–D) are listed on the right, while the strains previously reported and newly found are also indicated. Each OUT is named as its insect host (fig wasp) name followed by the fig name (FA: *Ficus auriculata*, FH: *Ficus hispida*, and FB: *Ficus benjamina*), and the numbers at last mean different haplotypes of *wsp* sequences.

**FIGURE 2:**
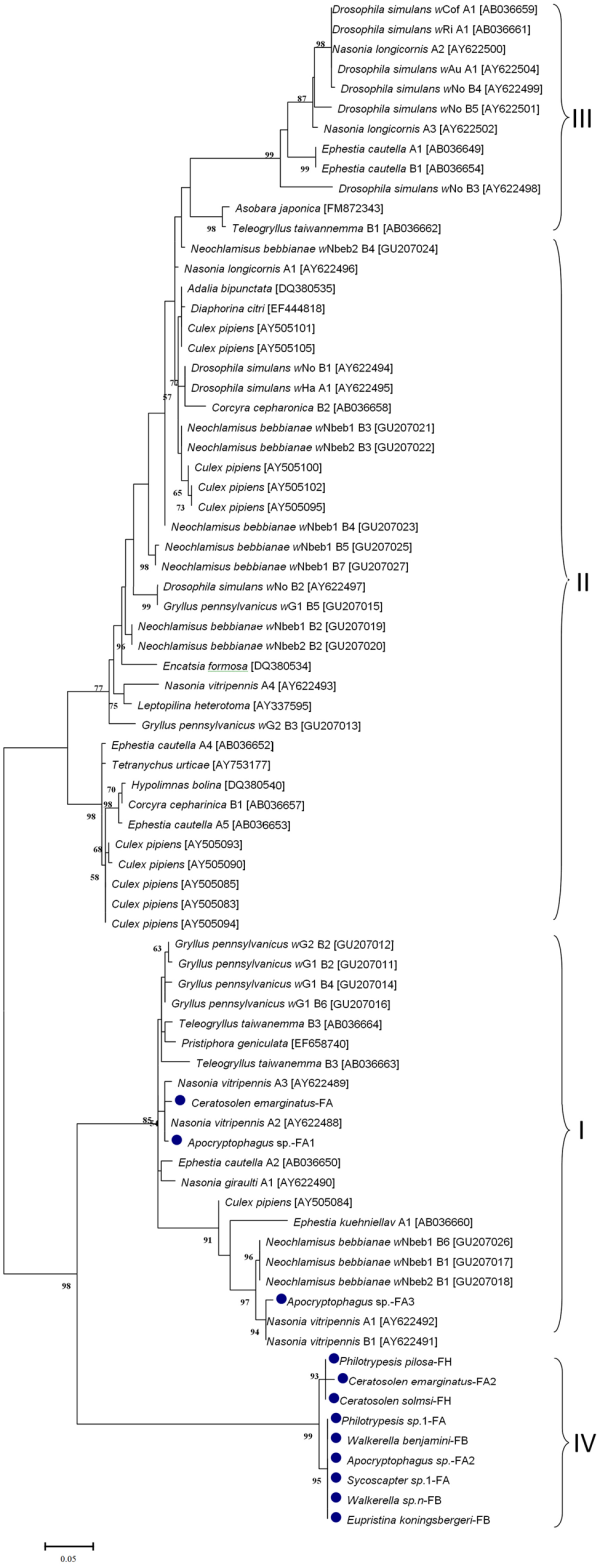
**Maximum likelihood phylogenetic tree of the bacteriophage *orf7* nucleotide sequences**. The sequences of fig wasps tested in our survey are marked with •. Some related sequences were downloaded from GenBank, and the accession IDs of the downloaded sequences are annotated with brackets. Different groups are indicated with I, II, III, IV. Samples tested in our survey are named by insect host name followed by the fig name (FA: *Ficus auriculata*, FH: *Ficus hispida*, FB: *Ficus benjamina*) and haplotype numbers.

**FIGURE 3:**
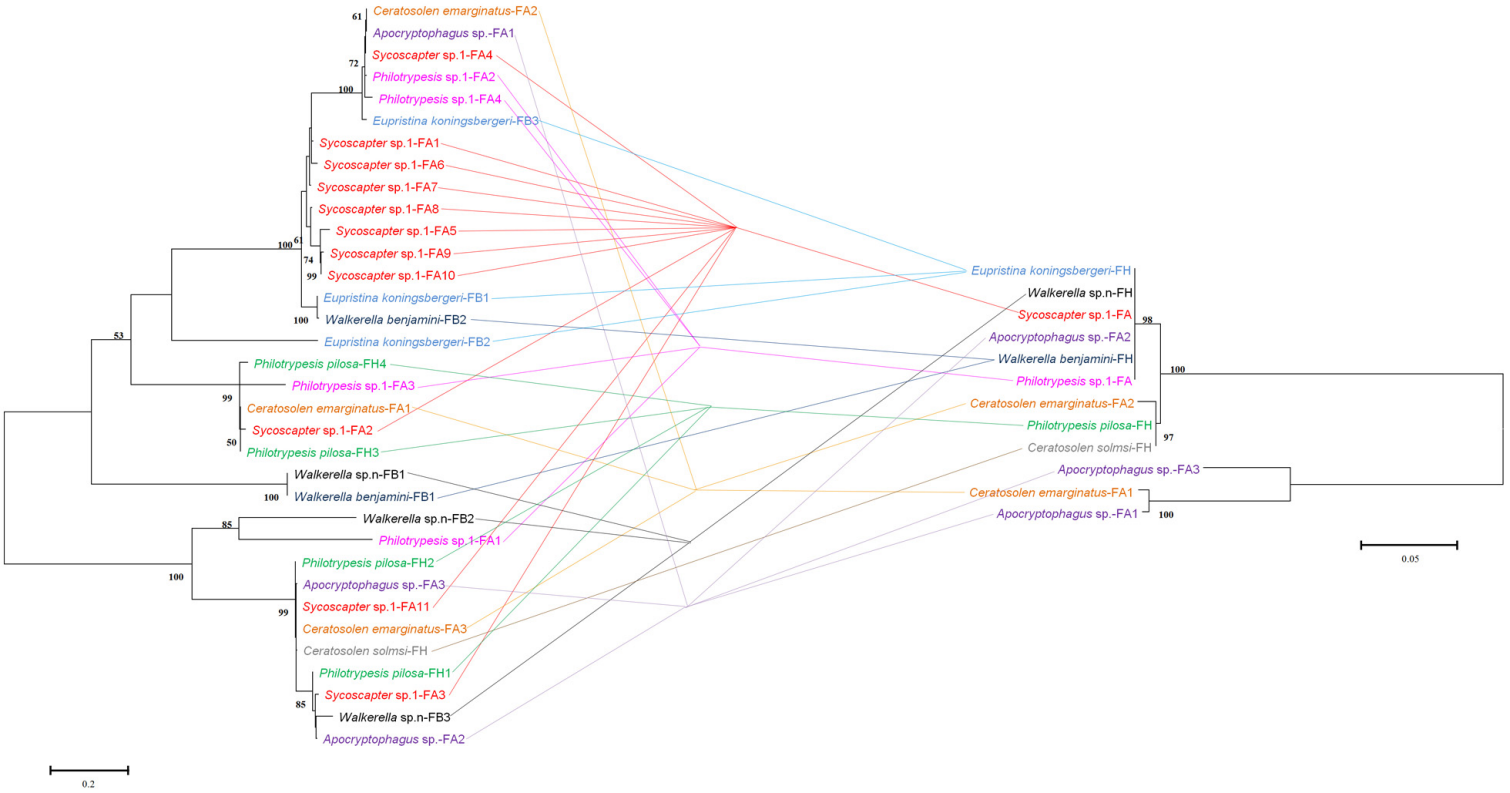
**Comparison between phylogenies of bacteriophage WO based on the *orf7* sequence (right) and *Wolbachia* based on the *wsp* sequence (left)**. Samples tested in our survey are named by insect host name followed by the fig name (FA: *Ficus auriculata*, FH: *Ficus hispida*, FB: *Ficus benjamina*) and haplotype numbers.

After removing repeated identical sequences within each species, 34 *wsp* haplotypes from 19 wasp species were obtained. Besides five common *Wolbachia* strains (*w*Haw, *w*Mel, *w*Uni, *w*Mors, and *w*Con), three other *Wolbachia* strains were first detected in fig wasps, named *w*Fig-1, *w*Fig-2 and *w*Fig-3, respectively (**Figure 1**). *w*Con belonged to *Wolbachia* supergroup B, while all the others belonged to *Wolbachia* supergroup A. *w*Haw and *w*Mel were widely distributed in *F. hispida* and *F. auriculata*, while *w*Con were only detected in *F. benjamina.* Among nine *Wolbachia*-infected species, *Ceratosolen solmsi* from *F. hispida* was infected by *w*Haw only and *Walkerella beniamini* from *F. benjamina* were double infected, while all the other seven fig species (*P. pilosa* from *F. hispida*, *Ceratosolen emarginatus*, *Apocryptophagus* sp., *Philotrypesis* sp.1, and *Sycoscapter* sp.1 from *F. auriculata*, and *Euprisina koningsbergeri* and *Walkerella* sp. from *F. benjamina*) were multiple infected. Noteworthy, *Sycoscapter* sp.1 from *F. auriculata* were infected by up to six different bacteria.

### WO Infection Patterns Among *Wolbachia* Strains Associated with Fig Wasps

No bacteriophage WO was detected in fig wasps that were not infected with *Wolbachia*, as has been reported in previous surveys (Fujii et al., 2004; Braquart-Varnier et al., 2005; Sanogo et al., 2005; Gavotte et al., 2007). Among the 23 fig wasp species tested, only 39% (9/23) were found to harbor phages. Importantly, all *Wolbachia*-infected fig wasps harbored phage WO. Notably, only three of 15 fig wasp species infected by *Wolbachia* harbored WO in *F. benjamina*, which was obviously lower than the infection rate in the other two fig species. Among the WO-infected species, most (7/9) had 100% infection rates, with the exception of *Walkerella beniamini* (42%) and *Walkerella* sp.n (22%) from *F. benjamina*. All the pollinators (3/3) were infected by WO, while only 30% (6/20) nonpollinators harbored phage WO.

Twelve *orf7* sequences were gained from the nine phage-harbored fig wasps. Most fig wasps harbored only one bacteriophage WO type while *Ceratosolen emarginatus* and *Apocryptophagus* sp., both of which were from *Ficus auriculata*, harbored two and three WO types, respectively. *Eupristina koningsbergeri*, *Walkerella benjamini*, and *Walkerella* sp.n from *F. benjamina*, *Philotrypesis* sp.1, *Sycoscapter* sp.1 and *Apocryptophagus* sp. from *F. auriculata* shared one common WO type, while the other two fig wasps from *F. hispida*, *Ceratosolen solmsi* and *Philotrypesis pilosa* shared another WO type.

Interestingly, the number and type of WO infections was not related to the *Wolbachia* host. On one hand, the same number of *Wolbachia* strains that infected different fig wasps harbored different WO types. For example, *Ceratosolen emarginatus*, which was infected by three different *Wolbachia* strains, harbored two types of WO phages, while *Apocryptophagus* sp., which was also infected by three different *Wolbachia* strains, harbored three different WO. On the other hand, one WO phage type could be found in many different *Wolbachia* strains. For example, *Sycoscapter* sp.1, which was infected by up to six different *Wolbachia* strains, was detected to harbor only one WO type.

### Bacteriophage WO Phylogeny

The phylogenetic ML tree of phage WO *orf7* sequences of fig wasps and 21 other insect species was constructed (**Figure 2**). Interestingly, most *orf7* fragments from fig wasps tested in our survey bunched together, belonging to one clade (group IV), only three (two from *Apocryptophagus* sp. and one from *Ceratosolen emarginatus*) *orf7* sequences located in other clades (group I, II, III) constituted by *orf7* sequences from the other 21insect species. The profile of the ML tree exhibited strong insect host specificity and suggested that WO in fig wasps had a special origin, which was quite different from previously surveyed insects from open and semi-open environments.

### Phage and *Wolbachia* Horizontal Transfer

**FIGURE 4:**
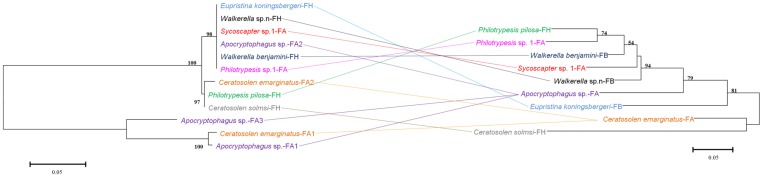
**Comparison between phylogenies of bacteriophage WO based on the *orf7* sequence (left) and fig wasps based on the *COI* sequence (right)**. Samples tested in our survey are named by insect host name followed by the fig name (FA: *Ficus auriculata*, FH: *Ficus hispida*, FB: *Ficus benjamina*), and haplotype numbers.

**FIGURE 5:**
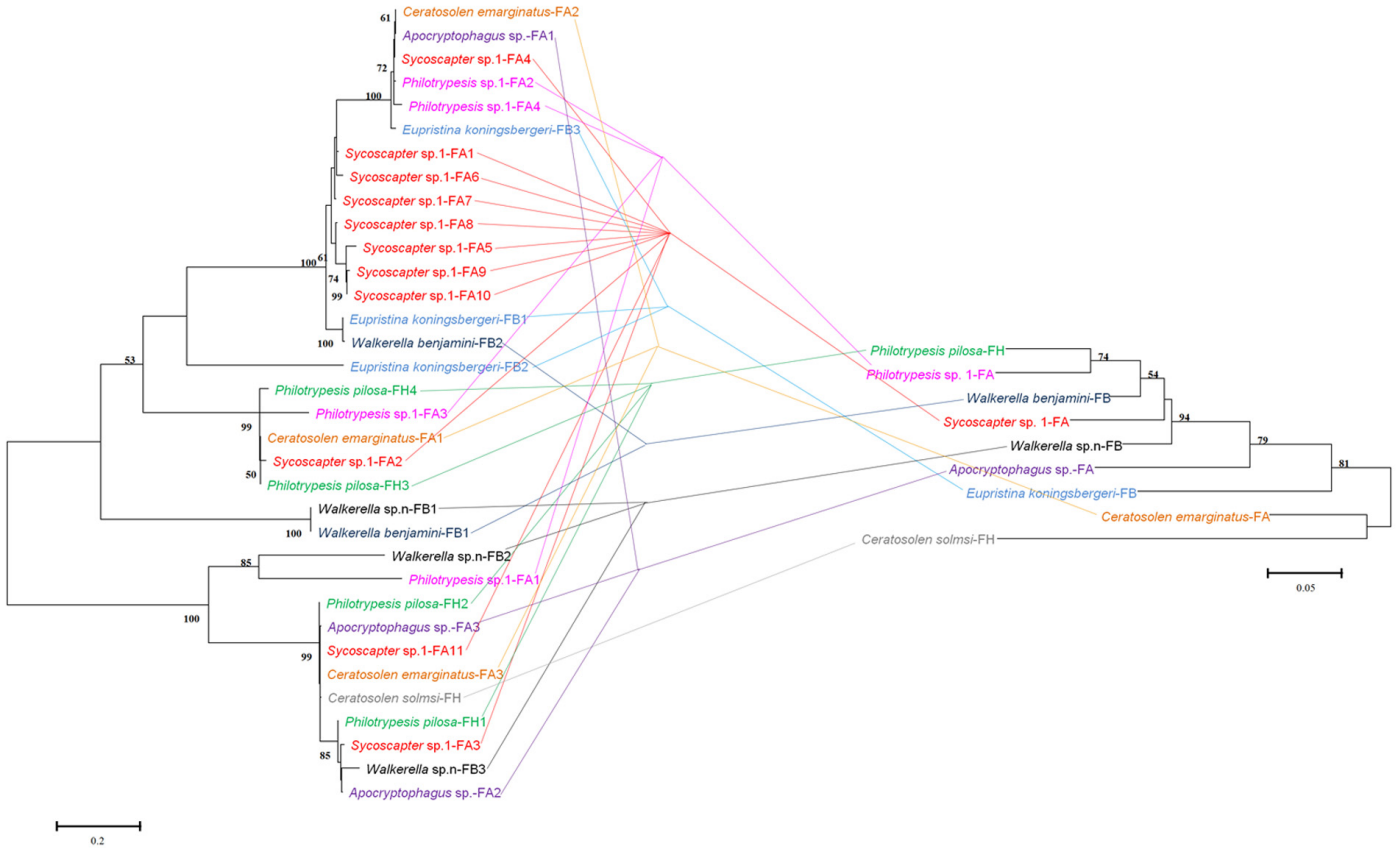
**Comparison between phylogenies of *Wolbachia* based on the *wsp* sequence (left) and fig wasps based on the *COI* sequence (right)**. Samples tested in our survey are named by insect host name followed by the fig name (FA: *Ficus auriculata*, FH: *Ficus hispida*, FB: *Ficus benjamina*) and haplotype numbers.

Comparisons of bacteriophage WO phylogeny, based on the *orf7* gene, and *Wolbachia* phylogeny, based on the *wsp* gene, were performed for 13 species infected by both WO and *Wolbachia* (**Figure 3**). Obviously, no congruence was found between phage WO and its host *Wolbachia* phylogenies, which indicated that phages do not cospeciate with their hosts. Moreover, there was also no congruence between phylogenies between phage WO and fig wasps (**Figure 4**), as well as between *Wolbachia* and its fig wasp hosts (**Figure 5**). No phylogenetic correlation was found between phages and bacteria, bacteria and insect hosts, or phages and insect hosts, as in some reported insects from open and semi-open environments (Masui et al., 2000; Bordenstein and Wernegreen, 2004; Gavotte et al., 2007). The absence of an evolutionary correlation between WO and *Wolbachia* and between WO and insect host phylogenies indicated that WO can be transferred horizontally by itself between different *Wolbachia* endosymbionts or even insect hosts.

In addition to this lack of congruence between WO and *Wolbachia*, we found other evidence of phage WO horizontal transfer. Distant phylogenetically related *Wolbachia* strains (supergroup A and B) shared the same WO type. For example, *Walkerella benjamini*, which was double infected by *Wolbachia* strains from different supergroups, harbored only one WO type. Moreover, different insect hosts from different figs (*Ficus auriculata* and *F. benjamina*), were found to harbor only one phage type.

## Discussion

Host–microbe–phage symbiosis comprises some of the most intimate and long-lasting associations on the planet. In order to determine the relationships between each pair of organisms, it is necessary to first determine the patterns of infection. In this study, we examined the relationships among *Wolbachia* and phage WO in fig wasp species within a closed system. Our data provided insights into the complex relationships of this symbiosis.

In this study, we report the detailed analysis and detection of *Wolbachia* and phage WO in 23 fig wasps from three different compact syconia. This system is notable because it is considered a closed community, different from other insects living in open or semi-open environments. In our study, the *Wolbachia* infection rate (83%) in our tested fig wasps was much higher than fig wasps in Australia (67%) and Panama (59%) (Shoemaker et al., 2002; Haine and Cook, 2005), which showed that fig wasps may have the highest known incidences of *Wolbachia* amongst all insects (Haine and Cook, 2005). As for bacteriophage WO, the infection rate in our tested fig wasps is 39%, which was unexpectedly lower than that in previous reports 89% (Chauvatcharin et al., 2006; Gavotte et al., 2007). The low WO infection rate (20%, 3/15) of fig wasps from *F. benjamina* and the high representation of fig wasps from *F. benjamina* in these samples (15/23) may contribute to the overall infection pattern. Interestingly, despite living in the same syconia, most *Wolbachia*-infected species did not harbor phage WO in *F. benjamina*. However, whether these fig wasps were infected previously or have never been infected is unclear. We suggest three possible explanations for this phenomenon. Firstly, the method used to screen for the presence of phage WO may not have detected all phage WO. Although several primer pairs were used to check the infection patterns repeatedly, the orf7F/R primers were not sufficiently degenerate to detect all *orf7* variants. Previously, only a single WO haplotype was detected in *D. simulans* infected with *Wolbachia* strain *w*Ri (Gavotte et al., 2007). However, the genome sequence confirmed four prophage copies in the genome (Klasson et al., 2009). Moreover, Metcalf proposed that single-gene PCR should not be used to rule out the presence of phage WO in *Wolbachia* (Metcalf et al., 2014). Second, the particular environment in which fig wasps live in may limit their physical interaction with the outside world. Unlike insects living open environments, chalcidoid wasps live almost all of their lives inside the syconia, which act as a physical barrier to prevent the insects from exchange with other insects. Third, there may be other unknown factors that isolate the insects or bacteria from phage WO infection. Therefore, future studies are needed to examine these possibilities.

Among the infections examined in this study, most bacteriophage WO types were first detected in fig wasps and showed strong insect host specificity based on the phylogeny of *orf7*, when considering 21 other insect species besides our fig wasps. However, it is not known what determines this specificity; specifically, we suspect that this specificity may be determined by a factor inherent in the chalcidoid and *Wolbachia* or within the unique living environment. As shown in the phylogenic tree, even species that were closely related with fig wasps exhibited substantial differences in *orf7* sequences. For example, *Nasonia* (*Nasonia vitripennis* and *N. longicornis*) and fig wasps are all hymenopterans, but their *orf7* genes are located distant from each other in the tree. Moreover, *Philotrypesis* sp.1, *Sycoscapter* sp.1, and *Nasonia* all belong to Pteromalidae, but their *orf7* genes are scattered in the tree. Therefore, the most probable explanation of specificity is the enclosed syconium in which the fig wasps live, which may result in a unique lineage for the bacteriophage WO. Importantly, the specificity is not limited to fig wasps living in one fig fruit, but is observed at the level of the fig wasp species.

Multiple infections are common in *Wolbachia*; however, six out of nine WO-infected species harbored only one phage type, which was confirmed by direct sequencing of the PCR product of *orf7* sequences, even when wasps were sampled from different syconia and different fig trees, indicating that they were not occasional infection events. In addition, the complete genome sequence of prophage WO (WOSol) in *Wolbachia* strain *w*Sol, which infected the fig wasps *Ceratosolen solmsi*, also showed the presence of only one WO phage in the *w*Sol genome (Wang et al., 2013). Furthermore, a survey of phage WO showed that 85% (28/34) of *Wolbachia* strains harbor only one or two different WO types (Gavotte et al., 2007).

*Wolbachia* spreads across hosts through both vertical and horizontal transfer. Vertical transmission is thought to be the predominant mode of *Wolbachia* transmission within a host (Werren et al., 2008), and horizontal transfer of *Wolbachia* has also been detected both within and among different host species in many cases (Baldo et al., 2006, 2008; Frost et al., 2010). Considerable horizontal transfer of *Wolbachia* has been detected within *F. benjamina*, and researchers have proposed that the syconium may provide a platform for horizontal transfer (Yang et al., 2012). We confirmed this by analyzing the discordance between the phylogenies of *Wolbachia* and fig wasps from different figs (**Figure 5**). Similarly, we found abundant horizontal transfer of phage WO in *Wolbachia* associated with fig wasps, although the exact horizontal transfer routes were uncertain. From our data and previous studies, there may be several possible mechanisms for such transfer. First, we observed multiple horizontal transfer of phage WO of *Wolbachia* in fig wasps from two pieces of evidence: 1) there was phylogenetic discordance between *Wolbachia* and phage WO (**Figures 2** and **3**) based on the *orf7* sequences, the same phage type was shared by phylogenetically distant *Wolbachia* strains of fig wasps from the same and different syconia. Thus, the horizontal transfer of both *Wolbachia* and WO was common in figs. Second, the horizontal transfer of WO was completely independent of the horizontal transfer of *Wolbachia*. If the horizontal transfer of WO depends on the transfer of *Wolbachia*, then the insects infected by the same *Wolbachia* strains would harbor the same WO types, and congruence between the two phylogenies would be found. However, this was not the case in our data. Instead, we found that WO could be successfully transferred by itself horizontally without its bacterial host between *Wolbachia* endosymbionts or insects. This has also been confirmed in other studies (Chafee et al., 2010). This independence may be possible because of the WO lysozymes, which could lyse bacterial cell walls (Fischetti, 2010). Third, the horizontal transfer of *Wolbachia* may facilitate the horizontal transfer of WO. The enclosed syconium provided a platform for horizontal transfer of the endosymbiont *Wolbachia*; this may also be beneficial for the transfer of the phage to some extent. Each of these potential mechanisms may contribute to the phage transfer.

Recently, genome sequence data shows that some mobile elements are present at sometimes high frequency in obligate intracellular bacteria, including *Rickettsia*, *Phytoplasma*, and *Wolbachia* (Cordaux et al., 2008; Baldridge et al., 2010; Chung et al., 2013). Bacteriophage WO is the only element that has been shown to move horizontally in obligate bacterial endosymbiont *Wolbachia* (Bordenstein and Wernegreen, 2004). WO is a dynamic element that has a marked effect on the genetic diversity of *Wolbachia* and could explain some of the interactions with *Wolbachia* genes and factors, without participating directly in the reproductive manipulations of *Wolbachia* induced in arthropods (Sanogo et al., 2005; Gavotte et al., 2007).

## Conclusion

We provided important insights into the horizontal transfer and interactions of fig wasps, *Wolbachia*, and phage WO in enclosed syconia. Some evidence of how bacteriophage WO work on *Wolbachia* or even insect hosts is another focus of our following research. This may facilitate the use of WO DNA-delivery vectors as tools for genetic manipulation of insects in the future.

## Author Contributions

NXW and DWH designed the study. NXW, SSJ, and HX performed the analyses. SSJ performed experiments. NXW, SSJ, YL, and DWH wrote the manuscript. All authors revised the manuscript and approved the final version.

## Conflict of Interest Statement

The authors declare that the research was conducted in the absence of any commercial or financial relationships that could be construed as a potential conflict of interest.
